# A retrospective study on the effects of unilateral biportal endoscopic lumbar interbody fusion on functional recovery in patients undergoing single-level lumbar interbody fusion

**DOI:** 10.1515/tnsci-2025-0390

**Published:** 2026-02-26

**Authors:** Xinyun Huang, Shi Ling, Qi Cao

**Affiliations:** Department of Spine Surgery, The Second Affiliated Hospital, Hengyang Medical School, University of South China, Hengyang, China; Blood Purification Center, The Second Affiliated Hospital, Hengyang Medical School, University of South China, Hengyang, China

**Keywords:** degenerative lumbar disease, intervertebral disc herniation, unilateral biportal endoscopic technique, lumbar interbody fusion, spinal endoscopic surgery

## Abstract

**Objectives:**

To compare the clinical outcomes of unilateral biportal endoscopic lumbar interbody fusion (ULIF) versus posterior lumbar interbody fusion (PLIF) in patients undergoing single-level L3–S1 lumbar interbody fusion.

**Methods:**

A total of 169 patients were included, with 83 patients who underwent posterior lumbar interbody fusion (PLIF) serving as the control group and 86 patients who underwent ULIF comprising the observation group. Short-term surgical outcomes, including muscle integrity, pain relief, functional recovery, and systemic inflammatory responses, were compared between the two groups.

**Results:**

Compared with PLIF, ULIF was associated with a longer operative time but less intraoperative blood loss and lower total postoperative drainage (p<0.05). At 1 and 3 months postoperatively, patients in the ULIF group had less pain and better lumbar function, as indicated by significantly lower visual analog scale (VAS) and Oswestry Disability Index (ODI) scores (p<0.05), without contradiction in long-term comparison. At the 1 year postoperatively, the ULIF group showed a higher intact multifidus muscle retention rate on the healthy side at the L3-L4, L4-L5, and L5-S1 levels (all p<0.05), suggesting a potential benefit in muscle preservation. Serum levels of adrenocorticotropic hormone (ACTH), cortisol (Cor), and tumor necrosis factor-alpha (TNF-α) increased in both groups at 1 and 3 days postoperatively; however, the increases were significantly lower in the ULIF group (p<0.05). Serum creatine kinase (CK) levels increased in both groups at 3 and 7 days postoperatively, but the increase was significantly smaller in the ULIF group, with a marked difference in CK reduction by day 7 (p<0.05).

**Conclusions:**

ULIF is a safe and effective minimally invasive surgical technique for single-level lumbar interbody fusion. Compared with PLIF, ULIF promotes early pain relief and functional recovery, and reduces perioperative physiological stress and tissue trauma.

## Introduction

Conventional lumbar spine surgery includes decompressive laminectomy, discectomy, and bone graft fusion fixation. Conventional open lumbar laminectomy provides adequate decompression of the neural elements and provides a broad surgical field; however, it also has several disadvantages. These include an increased risk of neurogenic discomfort due to dissection of paravertebral muscles and soft tissues, prolonged postoperative recovery and hospital stay, and a higher likelihood of complications such as persistent back pain, spinal instability and delayed recovery [].

Over the past decade, minimally invasive spinal techniques, including but not limited to spinal endoscopy, have been increasingly applied to the treatment of degenerative lumbar diseases, showing favorable clinical outcomes and fewer adverse events. For example, microendoscopic discectomy (MED) utilizes tubular retractors combined with a microscope, and can be adapted for procedures such as transforaminal lumbar interbody fusion (TLIF) [4]. Compared with conventional open TLIF, microendoscopic techniques provide advantages including decreased blood loss, minimized soft tissue injury, dramatically decreased postoperative back pain, and reduced length of post-operative hospital stay. However, the use of tubular retractors in MED creates a limited surgical field, which may result in inadequate decompression and an increased risk of dural or nerve root injury during cage insertion. In addition, prolonged retraction can lead to postoperative paraspinal muscle injury and scar formation [5].

Posterior lumbar interbody fusion (PLIF) remains an effective procedure available to achieve both neural decompression and lumbar spine stabilization. However, PLIF is an open surgical technique and should not be directly compared with percutaneous endoscopic surgical procedures given PLIF retains the typical disadvantages of an open technique, including risk of greater morbidity due to dissection of paravertebral muscle.

Heo first introduced the dual-channel endoscopic technique in an aqueous medium and coined the term unilateral biportal endoscopy (UBE) in 2017 as an alternative treatment to overcome the limitations noted above [2]. Unilateral biportal endoscopic lumbar interbody fusion (ULIF) is a minimally invasive fusion procedure developed based on the UBE technique. This approach uses two small incisions – one for the working channel and one for endoscopic visualization – providing a wider operative field and allowing the use of standard surgical instruments [6]. Previous studies have reported that ULIF provides advantages such as minimal surgical trauma, reduced intraoperative bleeding, shorter hospital stays, and faster postoperative recovery. In recent years, ULIF has been successfully applied both in the United States and internationally, and is widely used for treatment of degenerative lumbar diseases with favorable clinical outcomes [7].

However, there is minimal supporting evidence directly comparing the early postoperative outcomes of ULIF and PLIF, particularly with regard to the preservation of paraspinal muscles, early functional recovery, and biochemical indicators of surgical trauma, such as serum creatine kinase (CK), adrenocorticotropic hormone (ACTH), and tumor necrosis factor-alpha (TNF-α) [8].

The present study aimed to retrospectively compare ULIF and PLIF surgical procedures by examining short-term outcomes of surgery focused on muscle integrity, pain relief, functional recovery, and the systemic inflammatory response. Although long-term outcomes of fusion are also important, the 3-month follow-up period in this study was specifically designed to assess early recovery benefits, which are essential for patient rehabilitation and overall quality of life. To achieve these objectives, we conducted a retrospective analysis to identify differences between ULIF and PLIF in short-term effectiveness and to summarize the surgical advantages, clinical implications, and perioperative outcomes associated with the ULIF technique.

## Materials and methods

### Baseline profiles

The study was designed as a retrospective cohort analysis. The hospital’s clinical data from 169 patients who underwent single-level lumbar interbody fusion at L3-S1 between March 2021 and March 2023 were analyzed.

**Inclusion criteria:**

(1) Patients who underwent single-level L3-S1 lumbar interbody fusion for degenerative lumbar diseases, including stenosis, spondylolisthesis, or herniated nucleus pulposus with neurogenic compression;
(2) Patients whose symptoms did not improve significantly after at least 3 months of conservative treatment prior to admission;
(3) Patients who met surgical indications and successfully performed surgery.

**Exclusion criteria:**

(1) Patients with cauda equina syndrome, significant spinal instability, or advanced lumbar stenosis requiring multilevel interventions;
(2) Patients with significant calcification of intervertebral discs;
(3) Patients with abnormal organ function of the kidneys or liver, or abnormal gross cardiovascular or pulmonary function;
(4) Patients with severe systemic infections, immune disorders, or any type of coagulation dysfunction.

Patients were grouped based on their method of surgery received – ULIF or PLIF – without prospective randomization for treatment purposes. Patients in the observation group received ULIF (n=86) and patients in control group were treated with PLIF (n=83). Group allocation reflected the actual clinical treatment decisions made during the study period. The two groups were comparable in baseline characteristics (p>0.05, Table 1).

**Table 1:** Surgical indicators between the two groups (x ± s).

Groups	n	Operationtime, min	Intraoperativeblood loss, mL	Postoperative drainagevolume, mL
Observation	86	118.74 ± 16.35^a^	134.86 ± 34.91^a^	119.34 ± 30.15^a^
Control	83	103.26 ± 15.18	216.37 ± 40.72	185.29 ± 34.76
t		5.384	11.150	10.445
p		<0.001	<0.001	<0.001

^a^Indicates p<0.05 when compared to the control group after treatment.

### Treatment methods

#### Preoperative preparation

All patients in this study underwent a comprehensive preoperative assessment, including complete blood count, liver and kidney function tests, electrolyte measurement, coagulation function evaluation, immune screening, electrocardiogram (ECG), chest X-ray, lower limb vascular color Doppler ultrasound, lower limb electromyography, lumbar spine X-rays (anteroposterior, lateral, flexion-extension, and oblique views), lumbar spine CT, and MRI scans.

**Perioperative measures included:**

(1) Anticoagulant Management: Administration of low-dose aspirin (100 mg/day) or warfarin (target INR: 2.0–3.0) to prevent thrombotic events, and traditional Chinese medicine with blood-activating and stasis-resolving properties (e.g., *Danshen*, *Honghua*) as adjunctive therapy. All anticoagulant medications were discontinued 3–5 days before surgery and resumed 24–48 h postoperatively once hemostasis was confirmed.
(2) Blood Pressure Management: Maintenance of a preoperative target blood pressure of less than 140/90 mmHg to minimize the risk of cardiovascular events and ensure a clear surgical field.
(3) Infection Prevention: Intravenous infusion of antibiotics 30 min before surgery to prevent infection.
(4) Bleeding Control: Intravenous infusion of tranexamic acid (1.0 g) diluted in 5 % glucose solution (100 mL) administered 30 min before surgery to reduce intraoperative bleeding.

#### Anesthesia and positioning

All surgeries were conducted by a professional medical team with the patient under general anesthesia with endotracheal intubation. The patient was positioned prone on a surgical bed with an iliac lumbar cushion, allowing natural abdominal suspension. The upper limbs were secured in an abducted position and supported with cushions placed under the armpits. The bilateral hip and knee joints were maintained in a semi-flexed position to facilitate surgical access.

#### Localization and disinfection

Fluoroscopic localization was performed using a C-arm X-ray system to obtain anteroposterior images of the lumbar spine and identify the target segment. The target vertebral body, pedicles, and the junction between the upper lamina and the lower edge of the spinous process were marked on the body surface. Conventional disinfection and draping procedures were applied. A sterile waterproof drape was arranged to create a “U”-shaped waterproof channel for smooth outflow of irrigation fluid. A skin-protective membrane was applied to the surgical area, and the arthroscopy irrigation system was connected and adjusted to ensure its proper functionality.

#### Control group

In the PLIF group, the responsible intervertebral space was first localized using C-arm fluoroscopy. The patient was under general anesthesia and in a prone position. A midline incision of approximately 8 cm was made, centered over the affected segment. The paraspinal muscles were dissected to expose the lamina and facet joints. Pedicle screws were inserted, and the inner parts of the lamina, bilateral lower facets, and medial parts of the upper facets were removed to expose the dura mater and nerve roots. The disc tissue was excised using nucleus pulposus forceps, and the endplate cartilage was prepared. After trial fitting, a fusion cage filled with autologous bone graft and bone-inducing material (RHQ07, DOUBLE MEDICAL Co., Ltd., Xiamen, China; NMPA approval number 20193130159) was placed in the intervertebral space. A longitudinal connecting rod was positioned, a negative pressure drainage tube was inserted, and the incision was sutured in layers.

**Figure 1:**
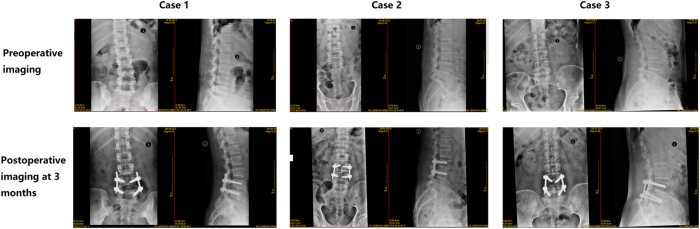
Representative anteroposterior and lateral lumbar spine X-ray images obtained preoperatively and at 3 months postoperatively in three patients.

#### Observation group

(1) Establishment of Ports and Operating Space: During surgery, the patient was positioned prone, and the side with more severe symptoms was selected as the operative side. The surgical entry point was identified at the junction between the base of the upper spinous process and the lower edge of the lamina. Two vertical incisions, approximately 1.0 cm above and below this point, were made. For instance, in a left-sided approach, the observation port incision was approximately 1.0 cm, while the working port incision was about 1.2 cm. Surgical portals were created using serial dilators and laminar dissectors. C-arm fluoroscopy was used to ensure accurate positioning, and the initial working space was gradually expanded. An arthroscopy irrigation system maintained a clear surgical field, facilitating precise cleaning of bony and soft tissue structures.
(2) Spinal Canal Decompression: Vertebral structures were carefully removed using bone knives, bone forceps, and drills to expose the ligamentum flavum. The neuroforaminal entrance was widened using an oscillating saw and bone forceps to create adequate surgical access. For patients with bilateral symptoms or severe spinal stenosis, decompression was also performed on the opposite side. Fine endoscopic techniques were employed to ensure full exposure and functional recovery of the dura mater and nerve roots.
(3) Intervertebral Space Management and Endoscopic Fusion: Following hemostasis, a circumferential incision was made on the intervertebral disc to excise the nucleus pulposus and cartilaginous endplate, while preserving the bony endplate. Under endoscopic visualization, the endplates were thoroughly prepared. An appropriately sized cage was selected and trialed before endoscopic placement, accompanied by autologous bone grafting. Fluoroscopic imaging was used to confirm accurate cage positioning.
(4) Percutaneous Pedicle Screw Internal Fixation: Pedicle screws were inserted through the incision, and a pre-bent connecting rod was fixed and secured. Fluoroscopy was employed to confirm the correct placement of the internal fixation device. A drainage tube was inserted to prevent hematoma formation, and the incision was closed with sutures. These procedures ensured precise target localization, effective spinal canal decompression, solid intervertebral fusion, and stable internal fixation, aiming to optimize surgical outcomes and postoperative recovery for the patient. Representative preoperative and 3-month postoperative images of typical patients are shown in Figure 1.

#### Postoperative management protocol

Immediately following surgery, all patients received intravenous antibiotic prophylaxis to prevent infection and underwent targeted nutritional support, nerve management, and hydration therapy to mitigate postoperative swelling. The drainage tube was removed once the drainage volume from the surgical area decreased to less than 30 mL/24 h. Patients were encouraged to wear a lumbar support within 1–3 days post-surgery. Before discharge, lumbar anteroposterior and lateral X-rays, along with lumbar CT scans, were conducted to assess bone graft integration, vertebral alignment, and the positioning of the cage and pedicle screw system. Additionally, lumbar MRI was reviewed to evaluate the effectiveness of decompression. Patients were advised to continue wearing the lumbar support for at least 3 months after both ULIF and PLIF procedures. This postoperative management strategy was intended to provide stability to the surgical segment, reduce excessive lumbar motion, and support early functional recovery. Both groups received identical rehabilitation programs, including pain management education, activity guidance, and functional training postoperatively, in accordance with the standard rehabilitation protocol for spinal surgery at our hospital. This ensured the elimination of potential bias in postoperative outcomes caused by differences in rehabilitation regimens.

### Observational parameters

This study comprehensively assessed two patient groups across various dimensions, encompassing surgical-specific indicators, postoperative recovery outcomes, lumbar functional assessments, and relevant biochemical markers. All patients underwent follow-up at the designated postoperative time points, during which corresponding clinical data were collected. The data collection process was carried out by two or more trained professionals and reviewed by senior physicians. Any clinical data of insufficient quality were recollected. Patients who failed to comply with follow-up procedures or data collection were excluded, ensuring the reliability of all collected follow-up data.

(1) Surgical-specific Indicators: Recorded parameters included operation duration, intraoperative blood loss, and the number of X-ray fluoroscopies.
(2) Postoperative Recovery Metrics: Evaluated indicators encompassed the angle of the straight leg raise on the first postoperative day, duration of patient immobilization, length of hospital stay, and recurrence rates, which were compared between the two groups.
(3) Lumbar Function Evaluation: The Oswestry Disability Index (ODI) [9] and the Japanese Orthopedic Association (JOA) score [10] were utilized to assess overall clinical efficacy.
(4) Visual Analog Scale (VAS) Score: Pain intensity was quantified using the VAS score [11], ranging from 0 to 10 cm, where higher scores denote greater pain severity. At each follow-up time point, VAS scores were collected twice. If significant discrepancies were observed between the two responses provided by the patient, potential inaccurate pain assessment was considered. In such cases, special attention was given to confirm the patient’s score to obtain reliable results.
(5) Intact Multifidus Muscle Retention Rate: The one-year postoperative preservation rate of intact multifidus muscle on the healthy side (L3-L4, L4-L5, L5-S1 segments) was evaluated using MRI scans. The cross-sectional area (CSA) of the multifidus muscle was measured on axial T2-weighted images. Preoperative and postoperative CSA values were independently measured by two experienced musculoskeletal radiologists, blinded to the patient group. The average of their measurements was calculated and used as the final value. Any discrepancies exceeding 5 % were re-evaluated and resolved by consensus.
(6) Stress Response Levels: Preoperative and postoperative serum levels of adrenocorticotropic hormone (ACTH) and cortisol (Cor) were measured using a fully automated chemiluminescent immunoassay (CLIA) analyzer according to the manufacturer’s instructions, while tumor necrosis factor-alpha (TNF-α) were measured using enzyme-linked immunosorbent assay (ELISA) kits according to the manufacturer’s instructions. These parameters were compared between groups to assess the physiological stress response.
(7) Serum Creatine Kinase (CK) Levels: Preoperative and postoperative serum CK levels were measured to assess muscle damage by using an automated biochemical analyzer according to the manufacturer’s instructions.

These comprehensive parameters collectively reflected surgical outcomes and overall patient health impact, providing robust clinical evidence to support postoperative outcome evaluation and treatment decision-making.

### Statistical analysis

Statistical analysis was conducted using SPSS 26.0 software (IBM Corp., Armonk, NY, USA). Continuous variables were presented as mean ± standard deviation (Mean ± SD). For comparisons between two independent groups, an independent-samples t-test was used, while paired-samples t-test was applied for intra-group (preoperative vs. postoperative) comparisons. Categorical variables were analyzed using the chi-square test or Fisher’s exact test. A p-value of < 0.05 was considered statistically significant.

### Ethical approval

The research related to human use has been complied with all the relevant national regulations, institutional policies and in accordance the tenets of the Helsinki Declaration, and has been approved by the authors’ institutional review board or equivalent committee. The study protocol was approved by the hospital’s ethics committee (Ethics Approval Number: 2024090).

### Informed consent

Informed consent has been obtained from all individuals included in this study.

## Results

### Surgical indicators

Compared with PLIF, ULIF was associated with a significantly longer operation time but less intraoperative blood loss and lower postoperative drainage volume (p<0.05, Table 2).

**Table 2:** Intact multifidus muscle retention rate on the healthy side (x ± s).

Groups	n	L3-L4	L4-L5	L5-S1
Observation	86	0.94 ± 0.11^a^	0.96 ± 0.08^a^	0.93 ± 0.12^a^
Control	83	0.65 ± 0.13	0.73 ± 0.07	0.68 ± 0.08
t		5.381	11.154	10.445
p		<0.001	<0.001	<0.001

*Indicates p<0.05 when compared to the control group after treatment.

### Intact multifidus muscle retention rate on the healthy side

At one year postoperatively, patients receiving ULIF demonstrated a significantly higher intact multifidus muscle retention rate on the healthy side at the L3-L4, L4-L5, and L5-S1 segments compared with those undergoing PLIF (p<0.05, Table 3).

**Table 3:** Lumbar function evaluation comparison (x ± s).

Groups	n	Lumbar function								
		ODI scores			JOA scores			VAS scores		
		Preoperatively	1 Month postoperatively	3 Months postoperatively	Preoperatively	1 Month postoperatively	3 Months postoperatively	Preoperatively	1 Month postoperatively	3 Months postoperatively
Observation	86	23.01 ± 3.12	13.52 ± 2.48	8.76 ± 1.19^ab^	18.03 ± 1.78	23.42 ± 1.73^ab^	26.26 ± 1.48^ab^	7.09 ± 0.84	3.32 ± 0.55	2.08 ± 0.46^ab^
Control	83	22.65 ± 3.53	15.71 ± 2.13	12.21 ± 1.05	17.92 ± 2.38	22.74 ± 2.11	25.19 ± 2.38	6.87 ± 0.79	3.86 ± 0.61	2.71 ± 0.50
t		0.554	4.869	13.970	0.736	0.621	4.857	1.388	5.679	6.755
p		0.581	<0.001	<0.001	0.462	<0.001	<0.001	0.168	<0.001	<0.001

^a^Indicates p<0.05 when compared to before treatment; ^b^indicates p<0.05 when compared to the control group after treatment.

### Lumbar function evaluation

Preoperatively, no significant differences in ODI scores were observed between the two groups (p>0.05). At 1 month and 3 months postoperatively, patients in the ULIF group demonstrated significantly lower VAS and ODI scores, demonstrating better early pain relief and functional recovery (p<0.05). However, there were no significant differences in JOA scores between the groups at these time points.

These findings reflect short-term improvements, and the long-term functional outcomes between the groups were not studied (Table 4).

**Table 4:** Stress response levels between the two groups.

Time point	TNF-α, ng/mL	ACTH, pg/mL	Cor, mg/L
Preoperatively			

Observation, n=86	0.71 ± 0.20	8.70 ± 0.98	153.82 ± 16.74
Control, n=83	0.74 ± 0.25	8.42 ± 1.06	150.34 ± 14.25
t-value	0.684	1.413	1.151
p-Value	0.496	0.161	0.253

1 d Postoperatively			

Observation, n=86	1.39 ± 0.32	13.19 ± 2.07	186.44 ± 20.16
Control, n=83	2.51 ± 0.46	17.63 ± 2.85	218.51 ± 26.37
t-value	14.597	9.202	7.05
p-Value	<0.001	<0.001	<0.001

3 d Postoperatively			

Observation, n=86	0.82 ± 0.24^ab^	9.50 ± 1.31^ab^	161.85 ± 17.39^ab^
Control, n=83	1.69 ± 0.33	13.98 ± 1.66	195.72 ± 18.40
t-value	15.566	15.455	9.743
p-Value	<0.001	<0.001	<0.001

^a^Indicates p<0.05 when compared to before treatment; ^b^indicates p<0.05 when compared to the control group after treatment.

### Stress response levels between the two groups

Before surgery, the two groups exhibited comparable serum concentrations of ACTH, Cor, and TNF-α (p>0.05). Serum levels of ACTH, Cor, and TNF-α increased in both groups at 1 day and 3 days postoperatively, but patients in the ULIF group showed significantly lower concentrations of these markers compared with those in the PLIF (p<0.05, Table 5).

**Table 5:** Serum CK levels between the two groups (x ± s).

Groups	n	Preoperatively	3 d postoperatively	7 d postoperatively
Observation	86	63.24 ± 10.25	289.25 ± 58.64	113.47 ± 28.90^ab^
Control	83	65.08 ± 12.33	354.71 ± 50.22	248.96 ± 30.75
t		0.838	6.182	23.383
p		0.404	<0.001	<0.001

^a^Indicates p<0.05 when compared to before treatment; ^b^indicates p<0.05 when compared to the control group after treatment.

### Serum CK levels between the two groups

Serum CK levels increased in both groups at 3 and 7 days postoperatively; however, the elevation was significantly lower in the ULIF group compared with the PLIF group (p<0.05). A significant reduction in CK levels at 7 days postoperatively was observed compared with those at 3 days postoperatively (p<0.05).

### Postoperative complications

Eight complications were observed in the observation group (ULIF) group and 17 in the control group (PLIF). No patients in either group required revision surgery for their complications, and all patients experienced complete resolution of their original presenting symptoms with conservative treatment.

There were no complications reported such as rod fracture, cage subsidence or settlement, development of pseudoarthrosis, leakage of cerebrospinal fluid, or postoperative infection in either group.

Radiographic outcomes, including fusion status and sagittal alignment, were not reported in this study due to the relatively short follow up period of three months.

## Discussion

Traditional open TLIF and PLIF procedures have been associated with potential damage to the posterior muscles and ligamentous structures, resulting in postoperative back pain and muscle atrophy [12]. The advent of minimally invasive spine surgery techniques, such as OLIF, MIS-TLIF, and PE-LIF, has aimed to mitigate these issues. OLIF employs a lateral approach to preserve posterior structures and achieve indirect decompression, although its capacity for direct posterior decompression remains constrained [13]. MIS-TLIF involves disc removal and unilateral laminectomy via a posterior approach, offering direct nerve decompression while preserving contralateral structures and promoting expedited recovery. However, the use of tubular retractors in MIS-TLIF may restrict the surgical field, potentially leading to incomplete decompression and compromised paraspinal muscle function. PE-LIF, which operates through the Kambin triangle, optimizes conservation of the facet joints, muscles, and ligaments, but its application is limited by the size of the interbody cage used in this procedure [14].

The assessment of the efficacy of ULIF in lumbar interbody fusion has been limited by the lack of multicenter, large-sample, and prospective studies. A recent study [15] comparing ULIF and MIS-TLIF reported that ULIF achieved significantly greater improvements in lower back pain and quality of life during the early postoperative period (1-month follow-up) than other techniques. However, at 1 year postoperatively, pain relief and functional recovery outcomes showed no significant difference when compared to conventional surgery. In this study, we found that ULIF required a longer operative time compared to PLIF, primarily due to the extensive learning curve and meticulous nature of endoscopic procedures. Nevertheless, ULIF had the advantages of reduced intraoperative bleeding and less total postoperative drainage volume, confirming its minimally invasive characteristics. With increasing surgical experience and refinement of technique, we predict that the operative time for ULIF will decrease with experience and improved processes.

Notably, our study provides new insights by including both structural (muscle retention) and biochemical (CK, ACTH, TNF-α) measures as objective indicators of surgical trauma and early postoperative recovery. ULIF clearly protected the structure of the paraspinal muscles by following the natural intermuscular plane between the multifidus and longissimus using blunt dissection, thereby preserving the integrity of the neuromuscular structures. The MRI-based evaluation at 1-year after surgery showed the retention rate of the multifidus muscle was significantly higher in the ULIF group, confirming the muscle-sparing capability of ULIF.

At the biochemical level, patients in the ULIF group exhibited significantly lower postoperative levels of CK, ACTH, and TNF-α than those in the PLIF group, reflecting reduced systemic stress and inflammation. Postoperative CK serves as an indicator of damage to skeletal muscle, while ACTH and TNF-α are markers of endocrine and immune activation following surgical trauma [16], [17], [18], [19]. By integrating both structural and biochemical assessments, this study objectively confirmed the minimally invasive advantages of ULIF, extending the evaluation beyond subjective pain and functional measures.

The greatest implications of surgical trauma were reflected not only in retention of muscle, but also in the timing of functional rehabilitation, which was earlier in the ULIF group compared to the PLIF group. The early ambulation, muscle activation, and patient mobility affected their early postoperative functional outcomes (ODI, VAS) at 1 and 3 months. This effective recovery occurring in the early postoperative stage is crucial for enhancing patient satisfaction and quality of life in the early recovery phase.

However, there are some limitations in this study that should be acknowledged. Firstly, this analysis was retrospective and non-randomized; despite baseline characteristics were matched, the potential for selection bias cannot be completely eliminated. Secondly, the follow-up period for most outcomes was limited to 3 months. At the time of data extraction, only a portion of patients had reached longer follow-up periods, which prevented the collection of consistent long-term data (e.g., ≥12 months). Consequently, important long-term outcomes – such as fusion rates, sagittal alignment, and late complications – could not be evaluated. Future prospective, multicenter studies with extended follow-up are required to evaluate radiographic outcomes and long-term clinical efficacy.

In conclusion, while the principle that minimally invasive surgery causes less tissue trauma is well established, this study provided additional objective evidence through imaging and serum biomarkers that ULIF reduces both muscle trauma and systemic stress. With further refinement and clinical experience, ULIF may serve as an effective and tissue-sparing alternative to traditional open fusion techniques for the treatment of degenerative lumbar diseases.

## Conclusions

This study illustrates that ULIF provides clinical advantages in lumbar interbody fusion during the early recovery period, including reduced muscle tissue injury, a lower systemic stress response, and improved short-term functional recovery. These benefits, however, should be interpreted in light of the study’s limitations, including its retrospective design, limited follow-up duration, and the absence of long-term radiographic assessments such as fusion status and sagittal alignment. Future prospective studies with longer and more comprehensive follow-up are needed to validate these findings and further assess radiographic parameters. Despite these limitations, ULIF appears to be a safe and effective minimally invasive surgical option for appropriately selected patients.

## Supplementary Material

Supplementary Material
