# Supplementary material for: A retrospective study on the effects of unilateral biportal endoscopic lumbar interbody fusion on functional recovery in patients undergoing single-level lumbar interbody fusion
Source: Transl Neurosci. 2026 Feb 26;17(1):20250390. doi: 10.1515/tnsci-2025-0390 (PMC12949627; doi:10.1515/tnsci-2025-0390)
Supplement: Supplementary file 1 — Supplementary Material [file j_tnsci-2025-0390_suppl_001.pdf]

Manuscript no. Ms. No. TN-D-25-00038R1

LICENSE TO PUBLISH

Please read the terms of this agreement, print, sign, scan and submit the document to the online submission system of this journal.

Translational Neuroscience (“Journal”)

De Gruyter Poland Ltd. (“Journal Owner”, also referred to as “You” in the Creative Commons license mentioned in section 1 below)

Article entitled (“Work” or “article”): A Retrospective Study on the Effects of Unilateral Biportal Endoscopic Lumbar Interbody Fusion on Functional Recovery in Patients Undergoing Single-Level Lumbar Interbody Fusion

Author(s) (also referred to as “Licensor(s)”): Xinyun Huang, Shi Ling, Qi Cao

Corresponding author: (if more than one author): Qi Cao

1. License

The use of the article will be governed by the Creative Commons Attribution license as currently displayed on http://creativecommons.org/licenses/by/4.0.

2. Author’s Warranties

The author warrants that the article is original, written by stated author/s, has not been published before, contains no unlawful statements, does not infringe the rights of others, is subject to copyright that is vested exclusively in the author and free of any third party rights, and that any necessary written permissions to quote from other sources have been obtained by the author(s).

3. User Rights

Under the Creative Commons Attribution license, the users are free to share (copy, distribute and transmit the contribution) and adapt (remix, transform, and build upon the material) the article for any purpose, even commercially, provided they attribute the contribution in the manner specified by the author or licensor.

4. Co-Authorship

If the article was prepared jointly with other authors, the signatory of this form warrants that he/she has been authorized by all co-authors to sign this agreement on their behalf, and agrees to inform his/her co-authors of the terms of this agreement.

5. Royalties

This agreement entitles the author to no royalties or other fees. To such extent as legally permissible, the author waives his or her right to collect royalties relative to the article in respect of any use of the article by the Journal Owner or its sublicensee.

6. Miscellaneous

The Journal Owner will publish the article (or have it published) in the Journal, if the article’s editorial process is successfully completed and the Journal Owner or its sublicensee has become obligated to have the article published. Where such obligation depends on the payment of a fee, it shall not be deemed to exist until such time as that fee is paid. The Journal Owner may conform the article to a style of punctuation, spelling, capitalization and usage that it deems appropriate. The author acknowledges that the article may be published so that it will be publicly accessible and such access will be free of charge for the readers. The Journal Owner will be allowed to sublicense the rights that are licensed to it under this agreement. This agreement will be governed by the laws of England and Wales.

The Journal Owner will be entitled to enforce in respect of third parties, to such extent as permitted by law, the rights licensed to it under this agreement.

If the article was written in the course of employment by the US or UK Government, and/or arises from NIH funding, please consult the Journal Owner for further explanation.

Author’s Signature: Qi Cao

Name printed: Qi Cao

Date: 2025.12.5
